# Matrix metalloproteinases (MMP) 3 and 9 as biomarkers of severity in COVID-19 patients

**DOI:** 10.1038/s41598-021-04677-8

**Published:** 2022-01-24

**Authors:** Monica Gelzo, Sara Cacciapuoti, Biagio Pinchera, Annunziata De Rosa, Gustavo Cernera, Filippo Scialò, Marika Comegna, Mauro Mormile, Gabriella Fabbrocini, Roberto Parrella, Gaetano Corso, Ivan Gentile, Giuseppe Castaldo

**Affiliations:** 1 CEINGE-Biotecnologie Avanzate, Scarl, via Gaetano Salvatore 486, 80145 Naples, Italy; 2 Dipartimento di Medicina Molecolare e Biotecnologie Mediche, Università di Napoli Federico II, Naples, Italy; 3 Dipartimento di Medicina Clinica e Chirurgia, Università di Napoli Federico II, Naples, Italy; 4 Divisione di Malattie Infettive Respiratorie, Dipartimento di Malattie Infettive e Emergenze Infettive, Ospedale Cotugno, AORN dei Colli, Naples, Italy; 5 Dipartimento di Medicina Traslazionale, Università della Campania L. Vanvitelli, Naples, Italy; 6 Dipartimento di Medicina Clinica e Sperimentale, Università di Foggia, Foggia, Italy

**Keywords:** Immunology, Biomarkers, Diseases, Medical research

## Abstract

The molecular basis of the wide clinical heterogeneity of Coronavirus disease 2019 (COVID-19) is still unknown. Matrix metalloproteinases (MMPs) may have a role in the lung damage and regeneration that occur in severe patients. We studied serum MMP3 and MMP9 as potential biomarkers of COVID-19 severity, in 108 hospitalized patients with different World Health Organization (WHO) severity stage and in 48 controls. At hospital admission, serum MMP3 was increased in COVID-19 patients with a significant trend along the progression of the WHO stage, while serum levels of MMP9 were significantly increased in COVID-19 patients with no correlation with disease severity. At 1 week from hospitalization, MMP3 was reduced, suggesting an early pathogenic role of the protein in lung inflammation, while MMP9 levels were further increased, indicating a late role of the protein in the inflammatory process, specifically during the repairing phase. Furthermore, serum MMP9 was positively correlated with serum interleukin-6, myeloperoxidase, and circulating neutrophils and monocytes number. In conclusion, serum MMP3 may help to early predict the severity of COVID-19 and both proteins, MMP3 and MMP9, may contribute to define severe COVID-19 patients that may benefit from a targeted therapy on MMPs.

## Introduction

The Coronavirus disease 2019 (COVID-19) shows a heterogeneous clinical expression, i.e., from asymptomatic or mild^1^ to severe forms^2^ with systemic inflammation and thromboembolic complications that led to acute respiratory distress syndrome (ARDS) and multi-organ failure. The molecular basis of the severe expression in a small number of COVID-19 patients is still undefined. The study of COVID-19 pathogenesis may help both to define prognostic biomarkers and to reveal altered pathways that could became the target of specific therapies.

Matrix metalloproteinases (MMPs) are a family of 24 zinc-dependent extracellular endopeptidases, which are widely expressed and involved in a myriad of biological processes. The main role of MMPs is the degradation of all components of extracellular matrix^3^, but these molecules are also involved in inflammation, modulating the synthesis and the release of cytokines and chemokines^4^, and in cell growth, proliferation, and remodeling^3^. Matrix MP3 is involved in the modulation of acute inflammation and ARDS of different etiology at lung level^3,5^. In fact, mice lacking MMP3 is prone to a less severe lung inflammatory injury^6^. Furthermore, it was found that serum levels of MMP-3 were related to the severity of pulmonary expression of COVID-19 patients^7^. For these reasons, the pharmacological inhibition of MMP3 was suggested as a potential therapeutic option in COVID-19 patients with severe ARDS^8^. Similarly, MMP9 is involved in lung inflammation and has a role in degrading the alveolar capillary barrier, promoting lung tissue damage, but it may have a role also in tissue repair^3^. Increased plasma levels of MMP9 were found in patients with severe ARDS^9^, and in 39 patients with COVID-19^10^. Another study described the immune-based signature of COVID-19 patients, relating serum MMP9 levels with the severity of COVID-19^11^.

To better define the role of MMP3 and MMP9 as biomarkers of COVID-19 outcome, we studied serum levels of these proteins in 108 patients with COVID-19 and in 48 healthy subjects. We related the levels of MMP3 and MMP9 to the disease stage, and to other biomarkers of inflammation.

## Results

Demographic data of 48 controls and 108 COVID-19 patients, classified on the base of the World Health Organization (WHO) stage, are reported in Table 1. No significant differences of age and gender were observed between controls and all patients (median age: 41; 44/108 males). Otherwise, the multiple comparison analysis showed significant differences among the control and the patient groups (p < 0.0001). In particular, the median age of patients and the male percentage were significantly (p < 0.01) higher in patients of WHO stage 4 and WHO stages 5–7 as compared with patients of WHO stage 3. Table 1 also reports the clinical data of COVID-19 patients. Among the comorbidities, we observed a higher percentage of patients with diabetes, hypertension, and obesity in advanced WHO stages. The other comorbidities, the hospitalization days, and the mortality agree with WHO stage classification of patients.

**Table 1:** Demographic and clinical data of controls and COVID-19 patients with different severity according to worst WHO stage.

	Controls	WHO 3	WHO 4	WHO 5–7
N	48	52	36	20
Age (years)	43 (33–61)	34 (28–41)^a^	51 (34–69)^b^	55 (48–77)^a,b^
Males, n (%)	27 (56)	8 (15)^a^	19 (53)^b^	17 (85)^a,b,c^
Diabetes, n (%)	–	5 (10)	13 (36)^b^	7 (35)^b^
Hypertension, n (%)	–	6 (12)	6 (17)	12 (60)^b,c^
Obesity, n (%)	–	5 (10)	6 (17)	7 (35)^b^
CAD, n (%)	–	4 (8)	3 (8)	3 (15)
CKD, n (%)	–	2 (4)	5 (14)	3 (15)
EI, n (%)	–	–	1 (3)	4 (20)^c^
ICU admission, n (%)	–	1 (2)	3 (8)	6 (30)^b,c^
ICU LOS (days)^d^	–	7	16 (11)	5 (4)
Hospital LOS (days)	–	6 (4–10)	14 (5–23)^b^	22 (17–29)^b^
Mortality, n (%)	–	–	–	7 (35)

Continuous data are reported as median and interquartile range. Categorical data are reported as frequency and percentage. ^a^p < 0.01, *versus* controls; ^b^p < 0.01, *versus* WHO 3; ^c^p < 0.01, *versus* WHO 4; ^d^data are reported as average (standard deviation). *CAD* coronary artery disease, *CKD* chronic kidney disease, *EI* endotracheal intubation, *ICU* intensive care unit, *LOS* length of stay.

**Figure 1:**
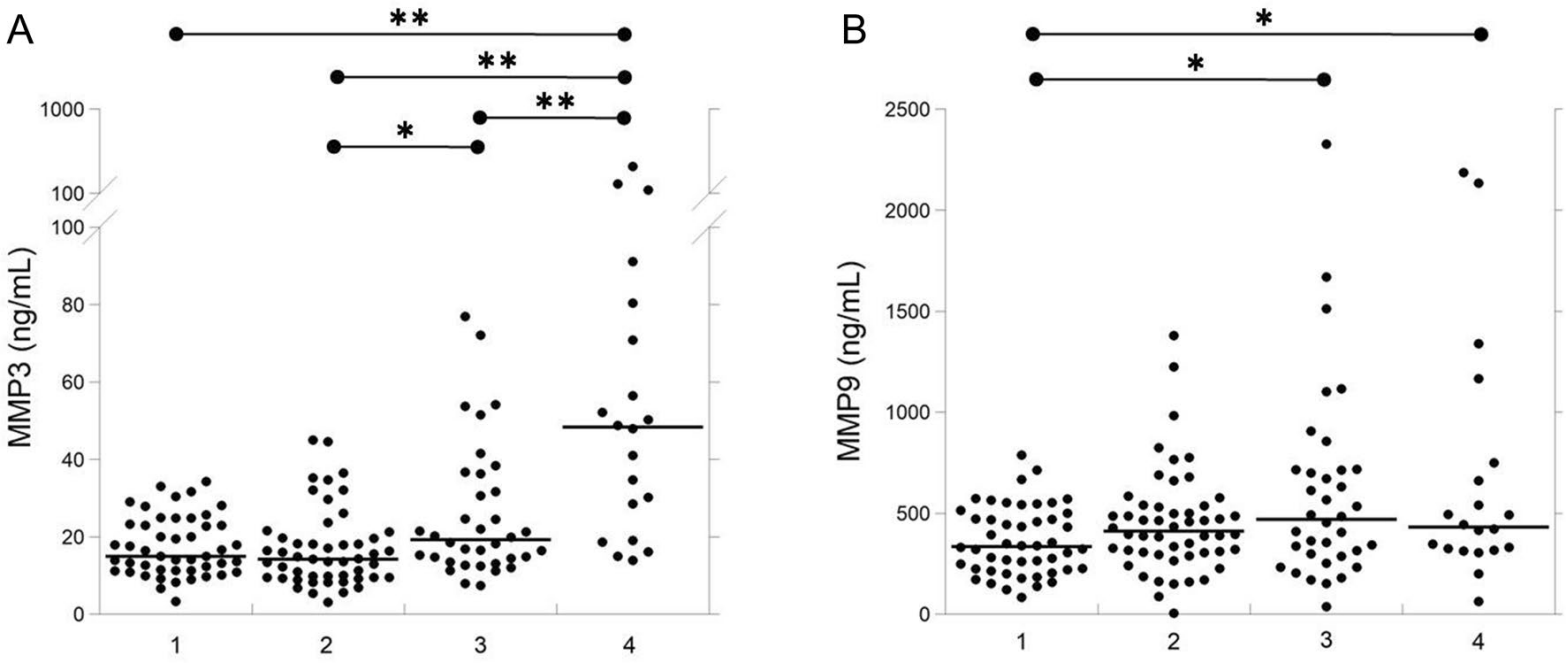
Dot-plots of serum levels of MMP3 (**A**) and MMP9 (**B**) in controls (lane 1) and in COVID-19 patients classified at WHO stage 3 (lane 2), WHO stage 4 (lane 3) and WHO stages 5–7 (lane 4). The black lines indicate the median value of data. *MMP* matrix metalloproteinase. *p < 0.01; **p < 0.001.

Figure 1 shows the levels of serum MMP3 and MMP9 in the control subjects and the COVID-19 patient subgroups. Table 2 shows the levels of serum MMP3 and MMP9 at admission in COVID-19 patients classified according to the WHO stage. Serum levels of MMP3 gradually increased along the WHO stages and resulted significantly (p < 0.01) higher in patients of WHO stage 4, as compared with patients of WHO stage 3, and significantly (p < 0.01) higher in patients of WHO stage 5–7, as compared with both patients of WHO stage 3 and 4. As compared with control group, the MMP9 serum levels of WHO stage 3 were increased not significantly, while the levels of WHO stage 4 and 5–7 were significantly higher. Serum levels of interleukin (IL)-6 were significantly higher in patients of WHO stage 4 and WHO stage 5–7 as compared with both controls and patients of WHO stage 3. The serum high-sensitivity C-reactive protein (hs-CRP) was significantly higher in all the three WHO stage groups as compared with controls, while no significant differences were observed among the WHO stages. The lymphocyte number was significantly lower in all the three WHO stage groups as compared with the controls. In addition, the patients of WHO stage 5–7 showed a significantly lower number of lymphocytes than the patients of WHO stage 3. The neutrophil number was significantly higher in WHO 5–7 group than both controls and WHO 3. Only the patients of WHO stage 4 showed a significantly higher number of monocytes as compared with the controls.

**Table 2:** Comparison of serum MMPs, inflammation markers and blood cells at admission in controls and COVID-19 patients with different severity according to worst WHO stage for each patient.

	Controls	WHO 3	WHO 4	WHO 5–7	Kruskal–Wallis
N	48	52	36	20	–
MMP3 (ng/mL)	15.6 (11.2–24.4)	14.5 (9.5–21.4)	19.1 (13.7–35.0)^b^	48.3 (21.4–77.9)^a,b,c^	< 0.0001
MMP9 (ng/mL)	333 (221–493)	411 (305–535)	468 (288–714)^a^	431 (318–727)^a^	0.048
IL-6 (pg/mL)	26.0 (19.8–41.9)	28.7 (23.7–44.3)	88.0 (29.2–256)^a,b^	37.4 (29.2–169)^a,b^	< 0.0001
IL-17A (pg/mL)	2.0 (2.0–2.4)	2.0 (2.0–3.9)	2.6 (2.0–4.9)	2.0 (2.0–3.5)	n.s
MPO (ng/mL)	413 (249–548)	371 (309–485)	482 (307–887)	352 (323–942)	n.s
hs-CRP (mg/L)	1.0 (0.6–2.1)	12.0 (5.5–23.0)^a^	15.5 (8.3–70.8)^a^	11.9 (2.3–75.5)^a^	< 0.0001
Lymphocytes (N/mmc)	1990 (1632–2300)	1332 (888–1851)^a^	1203 (812–1464)^a^	579 (377–1238)^a,b^	< 0.0001
Neutrophils (N/mmc)	4180 (2970–4840)	5642 (4249–6863)	5126 (3174–7762)	6298 (3029–9269)^a,b^	0.013
Monocytes (N/mmc)	380 (295–510)	503 (353–772)	578 (405–813)^a^	472 (280–827)	0.014

Median and interquartile range.

^a^p < 0.01, *versus* controls; ^b^p < 0.01, *versus* WHO 3; ^c^p < 0.01, *versus* WHO 4. *hs-CRP* high sensitivity C-reactive protein, *IL* interleukin, *MMP* matrix metalloproteinase, *MPO* myeloperoxidase, *n.s*. not significant.

**Figure 2:**
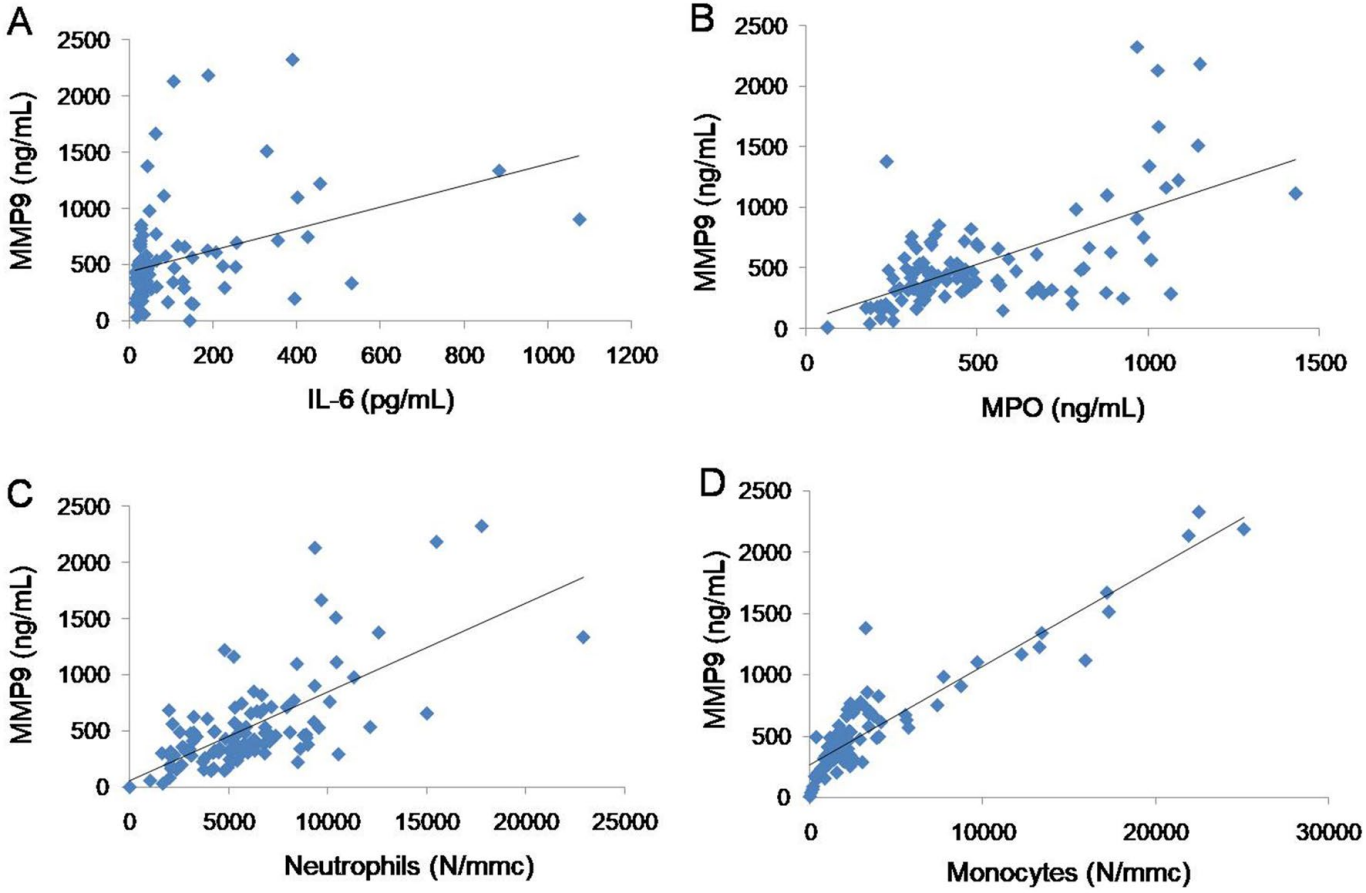
Spearman correlation analysis of serum MMP9 *versus* serum IL-6 (panel **A**; r_s_: 0.345, p < 0.0001), *versus* MPO (panel **B**; r_s_: 0.604, p < 0.0001), *versus* neutrophils (panel **C**; r_s_: 0.611, p < 0.0001), and *versus* monocytes (panel **D**; r_s_: 0.403, p < 0.0001). *IL* interleukin, *MMP* matrix metalloproteinase, *MPO* myeloperoxidase.

Spearman correlation analysis (Table 3) demonstrated that serum levels of MMP3 at admission were not significantly correlated with any other parameter, i.e., serum MMP9, interleukin (IL)-6 and IL-17, myeloperoxidase (MPO), hs-CRP, lymphocytes, neutrophils, and monocytes. While serum MMP9 at admission was significantly (p < 0.0001) and positively correlated with serum IL-6, MPO, and with the number of neutrophils and monocytes (Fig. 2).

**Table 3:** Correlations between serum MMPs and inflammation parameters in COVID-19 patients at admission.

	MMP3 (ng/mL)		MMP9 (ng/mL)	
	r_s_	p value	r_s_	p value
MMP9 (ng/mL)	0.081	0.407	**–**	**–**
IL-6 (pg/mL)	0.157	0.107	0.345	** < 0.0001**
IL-17A (pg/mL)	− 0.098	0.336	0.185	0.067
MPO (ng/mL)	0.051	0.530	0.604	** < 0.0001**
hs-CRP (mg/L)	− 0.056	0.567	0.168	0.081
Lymphocytes (N/mmc)	− 0.166	0.086	0.090	0.353
Neutrophils (N/mmc)	0.146	0.131	0.611	** < 0.0001**
Monocytes (N/mmc)	0.143	0.139	0.403	** < 0.0001**

*r*_*s*_: rho di Spearman. Significant values are reported in bold.

*hs-CRP* high sensitivity C-reactive protein, *IL* interleukin, *MPO* myeloperoxidase.

Among the 108 COVID-19 patients, 13 were treated with steroids and/or azithromycin before hospitalization. Thus, we assessed the effect of these drugs as well as the effect of age, gender, and comorbidities on serum levels of MMP3 and MMP9 by linear regression analysis. As shown in Table 4, the serum levels of MMP3 were positively related to the male gender and hypertension, while MMP9 was positively related to the age and the male gender of patients, diabetes, and the treatment with steroids.

**Table 4:** Linear regression analysis in COVID-19 patients.

	MMP3		MMP9	
	Slope	p value	Slope	p value
Age	0.131	0.089	0.221	**0.011**
Gender (male)	0.186	**0.027**	0.163	**0.046**
Diabetes	− 0.016	0.435	0.179	**0.032**
Hypertension	0.247	**0.005**	0.134	0.083
Obesity	− 0.041	0.337	0.100	0.152
Steroids^a^	− 0.002	0.494	0.154	**0.046**
Azithromycin^a^	− 0.003	0.490	− 0.159	0.050

^a^Among the 108 COVID-19 patients, 13 have been treated with steroids and/or azithromycin before hospitalization. Significant values are reported in bold.

Finally, for 52 COVID-19 patients we performed also a second sampling at 1 week from hospitalization. Table 5 compares the levels of serum MMP3 and MMP9 in these patients at admission (basal) and after 1 week of hospitalization. Serum levels of MMP3 did not change significantly after 1 week in none of the WHO subgroups. While the levels of MMP9, after 1 week, increased in all WHO subgroups, but were significantly higher in WHO stages 3 and 5–7.

**Table 5:** Comparison of serum MMPs in COVID-19 patients at hospital admission and after 1 week. Median and interquartile range.

	WHO 3 (n = 18)	WHO 4 (n = 24)	WHO 5–7 (n = 10)
**MMP3 (ng/mL)**			
Basal	18.8 (11.8–32.0)	20.0 (12.6–36.3)	45.6 (25.0–113.3)
After 1 week	15.0 (9.3–23.5)	19.6 (11.6–38.2)	57.5 (28.1–121.4)
p value^a^	n.s	n.s	n.s
**MMP9 (ng/mL)**			
Basal	314 (283–475)	429 (276–702)	456 (278–1406)
After 1 week	515 (334–603)	507 (287–792)	597 (343–1228)
p value^a^	**0.043**	n.s	**0.012**

^a^Wilcoxon signed-rank test. Significant values are reported in bold.* n.s*. not significant.

## Discussion

Serum levels of MMP3 were significantly higher in COVID-19 patients as compared with a control group of healthy subjects, and gradually increased with the WHO stage. While, serum MMP9 did not change with the progression of the WHO stage, although it was significantly higher in COVID-19 patients than in controls. Our study included hospitalized COVID-19 patients, all with pulmonary involvement, most of which (stages 4–7) requiring oxygen supplementation. The role of MMP3 in the pathogenesis of the lung damage during inflammation and the consequent tissue repair is still not completely defined^6^. On the other hand, the endothelial damage, which occurs at pulmonary level as well as in other districts, during severe COVID-19 infection and other pathologies, is well-known. The endothelial cells are the main producers of MMP3^12^. The production and secretion of MMP3 are triggered by inflammatory cytokines^13^, even if our data surprisingly excluded any correlation between serum MMP3 and serum IL-6 and IL-17 (that were increased in turn in patients with COVID-19). Furthermore, previous studies reported a higher activity of MMP3 in tissue in which there is a high number of neutrophils, while in inflamed tissues from rodent, genetically lacking MMP3, also the number of neutrophils is reduced^3^. Thus, it was suggested that MMP3 activity is necessary to help migration from vessels to the extracellular space of neutrophils, that in turn are the main responsible of tissue damage^6^. Moreover, it is surprising the lack of correlation that we found between serum MMP3 and both MPO and the number of circulating neutrophils, that in turn are increased in patients with COVID-19, particularly in advanced stages^14^. In any case, the increase of serum MMP3 during COVID-19 is an early event which confirms a major contribution of MMP3 during the initial phase of lung inflammation in the degradation of basement membranes^3^. In fact, MMP3 serum levels in our patients were significantly increased at admission and did not further increase after 1 week of hospitalization. Furthermore, in 12 patients tested at 1 month from the admission, the levels of serum MMP3 were invariably returned normal (data not shown).

The role of MMP9 during lung inflammation is not fully understood in turn. Its concentration in normal lung is very low, while it increases in different conditions like asthma, fibrosis, and COPD^15^. MMP9 production is induced by neutrophils and monocytes and accordingly we found a correlation of serum MMP9 *versus* serum MPO and the number of both neutrophils and monocytes. It has been observed that in lung tissue from COVID-19 patients the *MMP9* gene is up-regulated, and the protein contributes to the cytokine recruitment^16^. Our data indicate that the release of MMP9 during COVID-19 is not an early event in WHO 3 subgroup (see Table 5; n = 18), as compared with the controls. While, 1 week later the hospitalization, serum levels of MMP9 were increased in all WHO subgroups, as compared with the levels at admission. In addition, in most of the 12 patients, which were followed one month from admission, MMP9 levels were still increased. With these data it could be suggested that MMP9 might also be relevant to disease recovery.

The increase of serum/plasma levels of MMP3 and MMP9 during COVID-19 was already reported. In fact, a study described higher levels of serum MMP3 in 62 COVID-19 patients compared with a similar number of non-infected subjects, although no correlation between serum levels of the protein and the disease stage were assessed. Unlike our results, that study found a correlation between serum MMP3 and IL-6^7^. Similarly, an early increase of plasma MMP9 was found in 39 COVID-19 patients, also not assessing the correlation with the disease severity^10^, while another study on 175 patients reported, differently from us, a gradual increase of serum MMP9 with the severity of COVID-19^11^. Discordant levels of MMPs reported in the literature might be explained by use of plasma or serum. Higher MMPs levels have been observed in sera samples due to the release of them during the blood coagulation time.

Even if the role of the two MMPs in the pulmonary disease of COVID-19 patients is still not completely defined, the pharmacological inhibition of MMP3 was suggested as a potential therapeutic option in COVID-19 patients with ARDS^8^, while another paper suggested the repositioning therapy with aprothinin, an aspecific protease inhibitor, in COVID-19 patients with severe lung injury^17^. These therapies would have a role, considering that we demonstrated that steroids (commonly used in COVID-19 patients that require oxygen supplementation) do not modulate serum levels of MMP3. Interestingly, the distribution of MMP3 serum levels in COVID-19 patients are highly variable, thus we suggest that preliminary analysis of serum MMP3 at hospital admission may help to predict the severity of lung damage and to select COVID-19 patients that may benefit from targeted therapies.

A strength of this study is represented by new findings of an association of MMP3 *versus* COVID-19 severity and MMP9 *versus* inflammation. Kadry et al. showed that MMP3 activity are elevated in ARDS patients and the inhibition of MMP3 reduces the severity of bacterial lipopolysaccharide-induced ARDS in animal models^8^.

However, we identified some confounders that may represent a limitation of this study. In fact, the higher levels of MMPs in severely affected patients could depend also on the higher patient age, higher number of males, and some comorbidities (diabetes, hypertension, obesity) in these patients.

For the future studies, we are evaluating the possibility to treat patients with severe ARDS and elevated MMP3 levels by MMP-3 inhibitor or protease inhibitor in the very early stages of the disease.

## Methods

### Patients

The study was approved by the Ethical Committee of the University Federico II of Naples. All methods were performed in accordance with the relevant guidelines and regulations. Informed consent was obtained from all subjects. The lone exclusion criterion was the refusal or the impossibility to obtain the informed consent. We enrolled 108 adult hospitalized patients with a diagnosis of COVID-19 (SARS-CoV-2 infection). The 108 patients had a median age of 41 years (IQR: 32–61 years); 44/108 (41.0%) were males. The diagnosis of COVID-19 was confirmed by molecular analysis (RT-PCR) of the nasopharyngeal swab^18^. All the enrolled patients were classified on the basis of the seven ordinal scale made by the WHO-Research and Development Blueprint expert group and used in previous influenza studies^19,20^. According to this classification patients can be identified as: 1, not hospitalized with resumption of normal activities; 2, not hospitalized, but unable to resume normal activities; 3, hospitalized, not requiring supplemental oxygen; 4, hospitalized, requiring supplemental oxygen; 5, hospitalized, requiring nasal high-flow oxygen therapy, non-invasive mechanical ventilation, or both; 6, hospitalized, requiring extra corporeal membrane oxygenation, invasive mechanical ventilation, or both; and 7, death. For each patient, we considered the worst WHO stage during the infection. All biomarkers were tested at admission and, in 52 patients also at 1 week of hospitalization. In addition, we studied 48 healthy subjects as controls.

### Biochemical analyses

Serum samples were separated from blood cells after the collection in tubes without anticoagulant and stored at − 80 °C until analysis. Serum IL-6, MPO, MMP3 and MMP9 were analyzed by Human Magnetic Luminex Assay on Biorad Bio-Plex 100 system (Labospace s.r.l., Milan, Italy). Serum IL-17A was measured using specific human ELISA Max™ Set Deluxe kit (BioLegend, Inc., San Diego, USA), in accordance with the manufacturer's instructions. The serum hs-CRP was determined by a commercial kit (Abbott Diagnostics, Rome, Italy) and an automated biochemistry analyzer (Architect ci 16200 Integrated System, Abbott Diagnostics, Rome, Italy).

For cytometric analysis, the whole blood samples were collected in tubes containing EDTA and then analyzed by Facs Canto II (Becton Dickinson Italia, Milan, Italy). Lymphocytes, neutrophils, and monocytes were firstly separated on the basis of forward scatter and sideward scatter characteristics. In addition, the cells were gated with CD45 and sideward scatter^21^.

### Statistical analysis

Continuous data were reported as median and interquartile range (IQR). Statistical differences between three groups were assessed by Kruskal–Wallis test and Mann–Whitney U test as post-hoc test. Categorical data were reported as frequency and percentage. The chi-square test was used to compare the frequency of categorical variables between groups. Correlations between variables were evaluated by Spearman correlation analysis. Linear regression analysis was used to assess the effect of age, gender, comorbidities, and therapies (independent variables) on MMP3 and MMP9 (dependent variables) by stepwise method. Paired comparisons have been performed by Wilcoxon signed-rank test. Statistical analysis was performed by SPSS (version 27, IBM SPSS Statistics). Graphics have been performed by KaleidaGraph software (version 4.5.4, Synergy, Reading, PA, USA). p values < 0.05 were considered as significant.

## Data Availability

The datasets used and/or analysed during the current study are available from the corresponding author on reasonable request.

## Abbreviations

MMP: Matrix metalloproteinase
ARDS: Acute respiratory distress syndrome
IL: Interleukin
MPO: Myeloperoxidase
